# IL-10 from CD4^+^CD25^−^Foxp3^−^CD127^−^ Adaptive Regulatory T Cells Modulates Parasite Clearance and Pathology during Malaria Infection

**DOI:** 10.1371/journal.ppat.1000004

**Published:** 2008-02-29

**Authors:** Kevin N. Couper, Daniel G. Blount, Mark S. Wilson, Julius C. Hafalla, Yasmine Belkaid, Masahito Kamanaka, Richard A. Flavell, J. Brian de Souza, Eleanor M. Riley

**Affiliations:** 1 Immunology Unit, Department of Infectious and Tropical Diseases, London School of Hygiene and Tropical Medicine, London, United Kingdom; 2 Laboratory of Parasitic Diseases, National Institute of Allergy and Infectious Diseases, National Institutes of Health, Bethesda, Maryland, United States of America; 3 Section of Immunobiology, Yale University School of Medicine, New Haven, Connecticut, United States of America; 4 Department of Immunology and Molecular Pathology, University College London Medical School, London, United Kingdom; Center for Global Health and Diseases, United States of America

## Abstract

The outcome of malaria infection is determined, in part, by the balance of pro-inflammatory and regulatory immune responses. Failure to develop an effective pro-inflammatory response can lead to unrestricted parasite replication, whilst failure to regulate this response leads to the development of severe immunopathology. IL-10 and TGF-β are known to be important components of the regulatory response, but the cellular source of these cytokines is still unknown. Here we have examined the role of natural and adaptive regulatory T cells in the control of malaria infection and find that classical CD4^+^CD25^hi^ (and Foxp3^+^) regulatory T cells do not significantly influence the outcome of infections with the lethal (17XL) strain of *Plasmodium yoelii* (PyL). In contrast, we find that adaptive IL-10-producing, CD4^+^ T cells (which are CD25^−^, Foxp3^−^, and CD127^−^ and do not produce Th1, Th2, or Th17 associated cytokines) that are generated during both PyL and non-lethal *P. yoelii 17X* (PyNL) infections are able to down-regulate pro-inflammatory responses and impede parasite clearance. In summary, we have identified a population of induced Foxp3^−^ regulatory (Tr1) T cells, characterised by production of IL-10 and down regulation of IL-7Rα, that modulates the inflammatory response to malaria.

## Author Summary

Much of the pathology of malaria infection is due to an excessive inflammatory response to the parasite. The regulatory cytokine IL-10 is known to control inflammation during malaria infections and thus protect against immunopathology, but, in so doing, it reduces the effectiveness of other immune mechanisms which remove the parasites. In order to try to dissociate these two effects of IL-10, to allow simultaneous control of infection and avoidance of pathology, we need a better understanding of the processes leading to IL-10 production, the timing of its production, and the cells that produce it. In this study we have found that the major source of IL-10 during malaria (*Plasmodium yoelii*) infection is adaptive regulatory CD4^+^ T cells. This population is distinct from natural regulatory T cells and classical effector T cells. IL-10 derived from these adaptive CD4^+^ T cells prevents hepatic immunopathology but also suppresses the effector T cell response, preventing parasite clearance. Further work is now required to determine how these two key cell types (anti-parasitic effector T cells and IL-10-producing regulatory T cells) are induced, so that vaccines can be designed that will induce optimal numbers of each cell type at appropriate stages of the infection.

## Introduction

The erythrocytic stage of malaria infection is characterised by the development of strong pro-inflammatory immune responses which, although required to control parasite replication and promote clearance of infected erythrocytes, must be tightly regulated to prevent the immune-mediated pathology which is integral to the development of the severe complications of infection in humans and in a number of well-characterised animal models [1]–[3]. Previous studies have highlighted important roles for IL-10 and TGF-β in regulating the pro-inflammatory response during malaria infection [4]–[11]. Thus, although IL-10^−/−^ and TGF-β-depleted mice are able to control parasite replication during *P. chabaudi* AS infection as effectively as WT mice, unlike WT mice they develop severe TNF-mediated pathologies which are typically fatal [4], [9]–[11]. Similarly, IL-10 can prevent the onset of cerebral malaria in *P. berghei* ANKA-infected mice [8].

However, the exact role of IL-10 and TGF-β appears to vary between infections with different malaria species and strains, depending on the timing of cytokine production in relation to disease progression. Thus, production of TGF-β and IL-10 during the first few days of a lethal *P. yoelii* 17XL (PyL) infection is associated with inhibition of pro-inflammatory responses, rapidly escalating parasitaemia and death [5],[7]. In contrast, mice infected with the non-lethal variant (*P. yoelii* 17X; PyNL) produce no or only low levels of TGF-β and IL-10 during early acute infection and eventually control their parasitaemia [5]. Blockade of IL-10R signalling in combination with anti-TGF-β treatment restores the type-1 immune response during lethal *P. yoelii* infection, and a proportion of infected animals are able to control their infections and survive [5]. Moreover, splenocytes from susceptible BALB/c mice, but not resistant DBA/2 mice, infected with PyNL produce IL-10 and TGF-beta during the early acute stage of infection, which is associated with an increase in the proportion of splenic CD25^+^ CD4 T cells [12]. Taken together, these studies demonstrate a causal role for immunoregulatory cytokines in suppressing parasite clearance mechanisms.

In accordance with these findings, a study by Hisaeda and colleagues indicated that differential activation of natural regulatory T cells (nTreg) may account for the differing virulence of *P. yoelii* strains, since depletion of CD4^+^CD25^hi^ T cells (with anti-CD25 antibody) prior to infection converted PyL from a rapidly lethal infection into a resolving infection but had no effect on the course of PyNL infection [13]. Although first identified as cells that limit autoimmune pro-inflammatory responses [14], nTreg (defined by expression of CD4, the transcription factor Foxp3 and high levels of CD25) have since been shown to regulate the immune response in a number infections including *Leishmania spp* infections, *Mycobacterium tuberculosis* and helminth infections [15]–[18], mediating their effects either via direct cell contact or by release of cytokines. However, it is now becoming apparent that both adaptive (Foxp3^−^) regulatory T cell populations and classical T-bet expressing Th1 cells also play crucial immunoregulatory roles during infection and mediate their effects through secretion of IL-10 [19]–[21].

In this study we have examined the generation and function of both nTreg and adaptive IL-10-secreting T cells during malaria infection. We observe equivalent expansion of natural Foxp3^+^ regulatory T cells during both lethal and non-lethal *P. yoelii* infections but, using either anti-CD25 treatment or adoptive transfer of purified CD25^hi^/Foxp3^+^ nTreg or CD25^−^/Foxp3^−^ non-Treg T cell populations, we find no role for nTreg during PyL infection. Conversely, we demonstrate that populations of adaptive regulatory CD4+ T cells, that are CD25^−^, Foxp3^−^ and CD127^−^, and which do not make IFN-γ, IL-4 or IL-17, develop during both PyL and PyNL infections. These cells inhibit parasite clearance but, importantly, also prevent the development of pathology via production of IL-10. These data are consistent with the notion that whilst endogenous populations of nTreg may be sufficient to prevent immune-mediated pathology during chronic infections which induce rather modest inflammatory responses, such as avirulent leishmania, tuberculosis or helminth infections, rapid induction of distinct populations of adaptive/Th1 CD4+ T cells producing IL-10 may be required to counter the powerful inflammatory signals provided by virulent, rapidly replicating pathogens.

## Results

### Course of infection with PyL and PyNL in C57BL/6 mice

**Figure 1:**
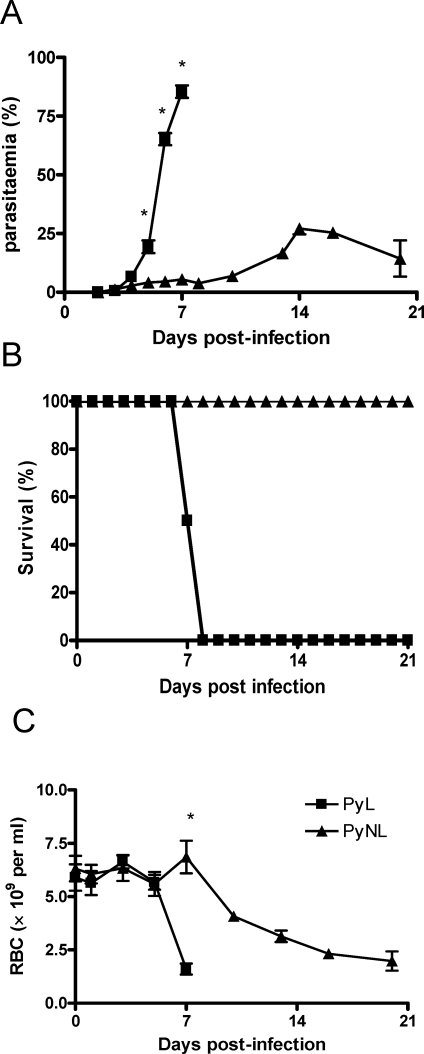
Course of infection of lethal (PyL) and non-lethal (PyNL) *P. yoelii* in C57BL/6 mice. C57BL/6 mice were infected i.v. with 10^4^ *P. yoelii* 17XL (PyL) or *P. yoelii* 17X (PyNL) parasites. The course of each infection was followed by monitoring (A) parasitaemia, (B) survival and(C) anaemia. 4–5 mice per group. Results are representative of 4 separate experiments. * = significant differences (p<0.05) between PyL and PyNL.

In accordance with previous observations [5],[22], infection of C57BL/6 mice with 10^4^ *P. yoelii* 17XL (PyL) parasites was associated with a rapid onset of fulminant parasitaemia (approaching 100% by day 7 pi) that was universally fatal (Figure 1A, B). In contrast, infection with 10^4^ *P. yoelii* 17X (NL) (PyNL) parasites led to a more gradual increase in parasitaemia with peak parasitaemia of approx. 30% on day 14 pi, before the infection eventually resolved. Significant differences in malaria-induced anaemia were also evident between lethal and non-lethal infections, with more rapid onset and increased severity of anaemia occurring in PyL-infected mice compared with PyNL-infected mice (Figure 1C).

### Similar expansion and activation of CD4^+^Foxp3^+^ regulatory T cells during PyL and PyNL infection

We have previously reported that simultaneous neutralisation of TGF-β and blockade of IL-10 signalling allows a proportion of PyL-infected mice to resolve their infections and survive [5], suggesting that active immune regulation/immune suppression occurs during PyL infection that inhibits optimal parasite control. In agreement with these observations, Kobayashi et al [7] have reported that IL-10 is produced very early during PyL (but not during PyNL) infection and Perry et al [23] have reported a switch from IL-12 (at day 3 pi) to IL-10 (at day 17 pi) production by splenic dendritic cells during the course of a non-lethal Py infection. These data are consistent with the hypothesis that protective pro-inflammatory responses develop during the acute phase of PyNL infection that limit parasite numbers, whereas an early anti-inflammatory cytokine response during the acute phase of PyL infection inhibits the development of protective immune responses. As CD4^+^CD25^+^ regulatory T cells (nTreg) have been reported to regulate immunity in a number of auto-immune and infectious diseases [14]–[18] and can exert their regulatory role through secretion of IL-10 and/or TGF-β we investigated, using intracellular staining for Foxp3 as well as transgenic Foxp3-GFP reporter mice [24], whether nTreg activation is correlated with the virulence of PyL infection.

**Figure 2:**
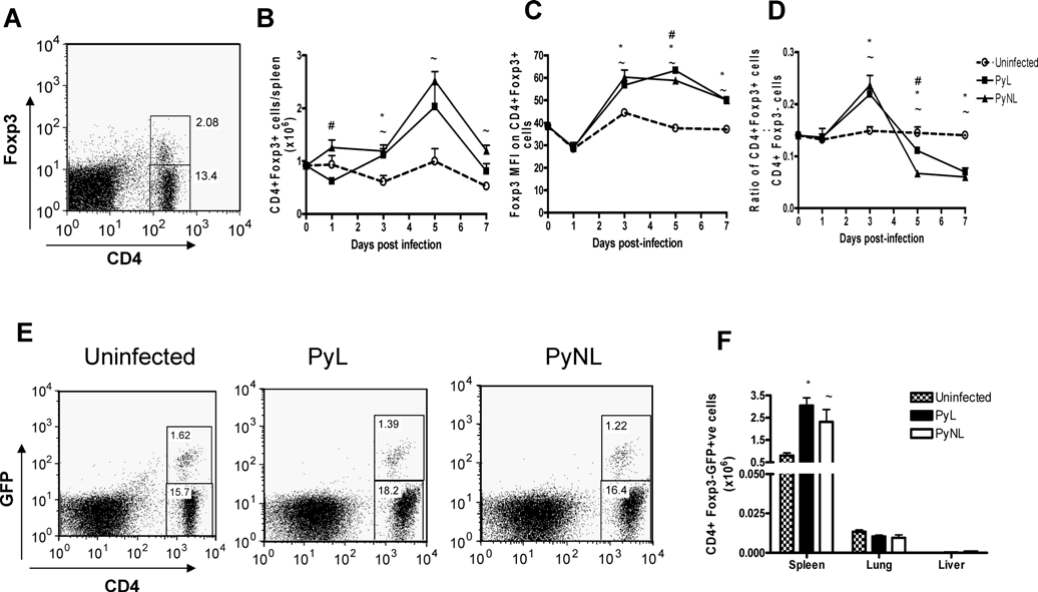
Expansion and activation of natural Foxp3^+^ regulatory T cell populations during PyL and PyNL infections. Numbers and activation status of Foxp3+ regulatory T cells were determined on various days post-infection with PyL or PyNL in (A–D) WT mice or (E–F) Foxp3-GFP transgenic mice. (A) Representative dot plot of intracellular staining in CD4+ T cells. On selected days post-infection with PyL or PYNL, splenic CD4^+^ lymphocytes were analysed for (B) numbers of Foxp3^+^ cells,(C) intensity (MFI) of Foxp3 staining and (D) ratio of Foxp3^+^ to Foxp3^−^ cells. (E) Representative dot plots showing GFP (Foxp3) expression in CD4+ splenocytes from 7 day PyL and PyNL-infected animals and uninfected controls. (F) Numbers of splenic GFP^+^ (Foxp3^+^) CD4^+^ regulatory T cells on day 5 post infection. 3–5 mice per group. Results are representative of 3 independent experiments. Symbols represent significant differences (p<0.05) between groups: # PyL vs PyNL; * PyL vs uninfected; ∼ PyNL vs uninfected.

CD4^+^ splenic lymphocytes from uninfected (control) mice, or from PyL- or PyNL- infected mice, were analysed for intracellular Foxp3 expression (Figure 2A) and the numbers of CD4^+^Foxp3^+^ cells, the expression levels of Foxp3 and the ratios of CD4^+^Foxp3^+^ (nTReg) to CD4^+^Foxp3^−^ (non-regulatory T cells) were assessed over the first 7 days pi (Figure 2B–D). In accordance with previous observations [25] a significant increase in the numbers of splenic CD4^+^Foxp3^+^ nTreg was observed during the first 5 days of PyL infection (Figure 2B) and this was accompanied by increased levels (MFI) of Foxp3 expression (Figure 2C) and a transient increase (on day 3pi) in the nTreg/non-Treg ratio (Figure 2D). However, almost identical changes in nTreg numbers and Foxp3 expression levels were observed in mice infected with PyNL, and there were no significant differences in any nTreg parameter between PyL-infected and PyNL-infected mice at any time up to 7 days pi, after which the PyL-infected mice succumbed to their infections.

Similar results were obtained with Foxp3-GFP reporter mice [24]. Importantly, the course of PyL and PyNL infections were equivalent in Foxp3-GFP mice and C57BL/6 mice (data not shown). A representative plot showing Foxp3-GFP expression in infected and uninfected mice is shown in Figure 2E. Numbers of CD4^+^GFP^+^ cells were significantly increased in the spleen on 5 day pi (Figure 2F) and on day 7 pi (data not shown) but did not differ significantly between PyL-infected and PyNL-infected mice. Finally, no significant differences were observed in expression of Foxp3 mRNA in CD4^+^ T cells purified from spleens of PyL and PyNL-infected mice on days 1, 3, 5 and 7 pi (data not shown).

### Neither CD25^hi^ nor Foxp3^+^ endogenous regulatory CD4^+^ T cells contribute to the virulence of lethal *P. yoelii* infection

**Figure 3:**
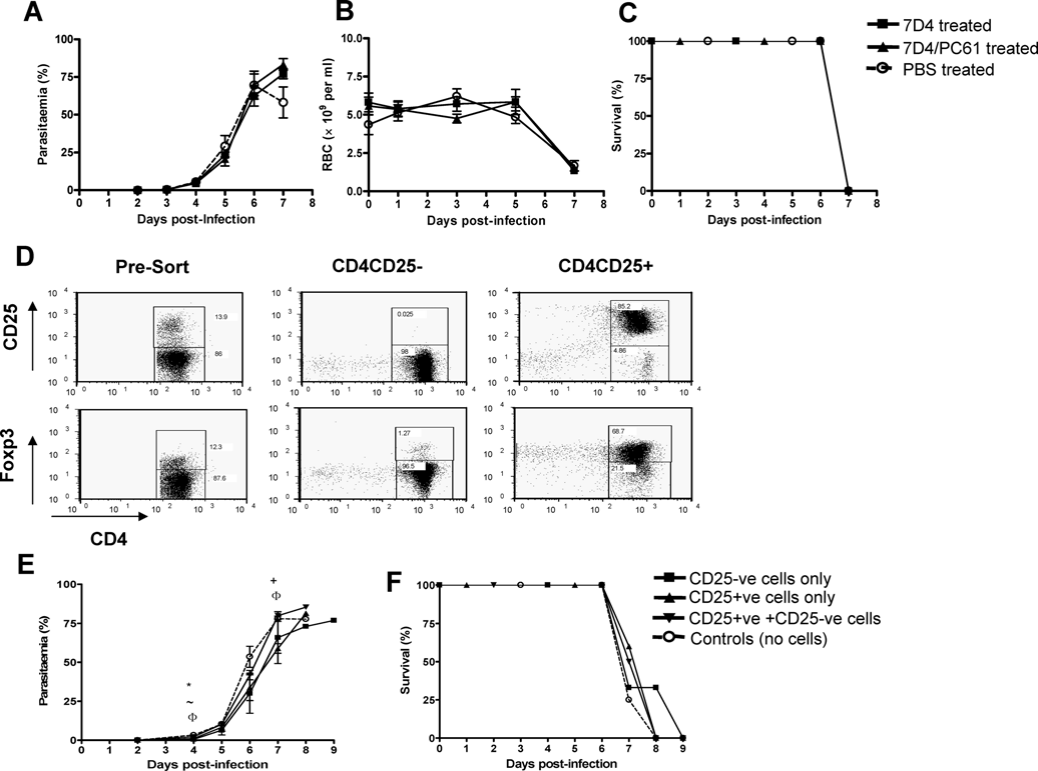
Natural Treg do not significantly contribute to the virulence of PyL infection. (A–C) Mice were given either a single dose of 0.75 mg 7D4 (IgM clone) or 0.25 mg 7D4 combined with 0.75 mg PC61 (IgG1 clone) on 3 days prior to infection with 10^4^ PyL pRBC. The effect of anti-CD25 treatment on the course of PyL infection was examined in (A–C) C57BL/6 mice by following (A) parasitaemia, (B) anaemia and (C) weight loss. Groups consisted of 3–5 mice and data are representative of 2 independent experiments. Symbols represent significant differences (p<0.05) between groups: (A–D) # 7D4 vs 7D4/PC61; * 7D4 vs PBS; ∼ 7D4/PC61 vs PBS; (G–H) # 7D4 vs 7D4/PC61; * 7D4 vs 7D4×3; ∼ 7D4 vs PBS; + 7D4/PC61 vs 7D4×3; Φ 7D4/PC61 vs PBS; Δ 7D4×3 vs PBS. (D–F) Naive CD4^+^CD25^−^ (non-Treg) and CD4^+^CD25^hi^ (Treg) cells were purified by flow cytometric cell sorting and adoptively transferred alone or at a 10∶1 ratio (non Treg∶Treg) into RAG-1^−/−^ mice prior to infection with PyL parasites. (D) shows the purity of sorted CD4^+^CD25^−^ and CD4^+^CD25^hi^ populations prior to adoptive transfer and the relative expression of Foxp3 within the purified populations. The affect of adoptive transfer on the course of infection was determined by monitoring (E) parasitaemia and (F) survival for the duration of the experiment. Groups consisted of 5 mice and the results are representative of 2 independent experiments. Symbols represent significant differences (p<0.05) between groups: # CD25^−^ vs CD25^+^; * CD25^−^ vs CD25^−^/ CD25^+^; ∼ CD25^−^ vs control; + CD25^+^ vs CD25^−^/ CD25^+^; Φ CD25^+^ vs control; Δ CD25^−^/ CD25^+^ vs control.

The similarity of the nTreg response during PyL and PyNL infections suggested that, in our hands, suppression of effector cell responses by nTreg was unlikely to explain the highly virulent nature of PyL infections. However, to formally test the role of nTreg, mice were treated with a cocktail of anti-CD25 antibodies (previously shown to give optimal depletion of CD4^+^CD25^hi^Foxp3^+^cells; 25) 3 days prior to infection with PyL (Figure 3). As previously reported [25], the 7D4 (IgM, anti-CD25) antibody substantially reduced the proportion of splenic CD25^+^ CD4 cells within 3 days (i.e day of infection) but CD25^+^ cells recovered to normal levels by day 4 pi (results not shown) and 7D4 treatment had no significant effect on the frequency of CD4^+^Foxp3^+^ve cells (results not shown). In contrast, PC61 (IgG anti-CD25) given in combination with 7D4 induced an approximately 50% reduction in the frequency of both CD25^+^ and Foxp3^+^ cells that was sustained throughout the 7 day infection period [25]. Nonetheless, neither 7D4 treatment nor combined 7D4+PC61 treatment significantly altered the course of parasitaemia, anaemia or survival of PyL infection in C57BL/6 mice (Figure 3A–C). As these observations contradict those of a similar published study [13] we considered whether some effect of natural T reg might be being masked by the rapidly ascending parasitaemia and early mortality associated with infection with 10^4^ PyL parasites. We therefore repeated the anti-CD25 antibody treatment in C57BL/6 mice infected with either a 10 fold lower dose of PyL parasites (10^3^ PyL) or with 10^4^ PyNL parasites. However, anti-CD25 antibody treatment did not alter the outcome of either of these infections (Figures S1 and S2) suggesting that natural T reg cells do not markedly influence *P. yoelii* infections in C57/BL6 mice.

It has been reported that regulatory T cell responses are more effective at limiting pro-inflammatory responses in BALB/c mice than in C57BL/6 mice [26]. Therefore, to determine whether mouse strain influences the outcome of anti-CD25 treatment during PyL infection, we repeated the CD25-depletion experiments in BALB/c mice and compared our depletion strategies (single dose of 7D4 or 7D4+PC61 given 3 days prior to infection) with a strategy previously shown to affect PyL infection [13], namely repeated injections of 7D4 antibody on days −3, −1 and 5 relative to PyL infection. Repeated administration of 7D4 did not increase either the duration of CD25^+^ T cell depletion or the extent of depletion of CD4^+^Foxp3^+^ve cells compared to the other treatment regimes (Figure S3). Consistent with this, repeated administration of 7D4 did not alter the course of PyL infection compared with single 7D4 administration or combined 7D4 and PC61 administration (Figure S3), and none of our CD25-depletion regimes had any effect on PyL infection in BALB/c mice (Figure S3).

It is becoming increasingly evident that anti-CD25 antibody treatment is not a specific or robust strategy to examine the importance of natural regulatory T cells during inflammatory episodes [25], [27]–[29]. CD25 expression is not limited to nTreg [24]. Moreover, depending on the precise protocol used, a variable but significant proportion of Foxp3^+^ cells escape depletion by anti-CD25 antibody. We therefore compared the outcome of PyL infection in RAG−/− mice reconstituted or not with purified naïve CD4^+^CD25^−^ (putative effector) T cells or a 10∶1 ratio of effector (CD4^+^CD25^−^) to nTreg (CD4^+^CD25^+^) cells. Furthermore, as nTreg can down-regulate NK cell responses [30], and as NK cells have previously been reported to play a protective role during malaria infection [31]–[33], we adoptively transferred CD4^+^CD25^+^ (nTreg) cells in the absence of CD4^+^CD25^−^ (effector) cells, to determine whether nTreg modulate innate immune responses during malaria infection. The proportion of Foxp3^+^ cells fell from 10–15% in unsorted CD4^+^ T cells to 1–2% in the CD25^−^CD4^+^ population, whereas CD25^+^ cells were highly enriched for Foxp3^+^ cells (70–80%; Figure 3D).

In accordance with our previous studies [22], we found that control (unreconstituted) RAG^−/−^ mice succumbed to PyL infection with the same kinetics as WT mice (compare Figure 3E, F with Figure 1). Furthermore, the course of infection was virtually indistinguishable in RAG^−/−^ mice reconstituted with CD4^+^CD25^−^, CD4^+^CD25^+^ or a 10∶1 ratio of CD4^+^CD25^−^/CD4^+^CD25^+^ T cells (Figure 3E, F). Thus, using two independent models of nTreg depletion, we have found no significant role for natural CD4^+^CD25^+^Foxp3^+^ regulatory T cells in suppression of anti-parasitic immunity during PyL infection in either C57BL/6 or BALB/c mice.

### PyL- and PyNL-induce IL-10 production from CD4^+^, CD25^−^, Foxp3^−^ T cells that express low levels of IL-7Rα

Having found no evidence that nTreg influence the outcome of PyL infection we next investigated the possibility that IL-10 producing CD4^+^ T cells (“adaptive” Treg or Tr1 cells) might be induced during PyL and/or PyNL infection that regulate parasite killing and/or pathology.

**Figure 4:**
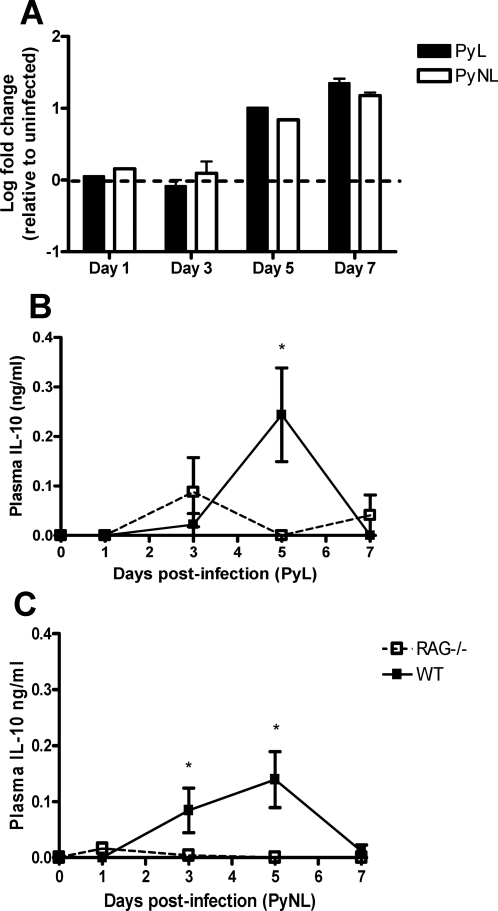
CD4^+^ T cells are a major source of IL-10 during both PyL and PyNL infection. (A) On selected days post-infection, CD4^+^ T cells were purified from PyL and PyNL infected mice by MACS sorting and levels of IL-10 mRNA were determined by real time PCR (Taqman) relative to the house keeping gene GAPDH. The results are shown as the fold change in expression relative to uninfected naïve CD4+ T cells. In separate experiments the levels of IL-10 in the plasma of WT and RAG-1^−/−^ mice were determined by ELISA on selected days of (B) PyL and (C) PyNL infection. Groups consisted of 3–5 mice and the results are representative of 2 separate experiments. For Taqman analysis, purified cells from several mice in each group were pooled; no significant differences between groups were identified. * indicates significant differences (p<0.05) between WT and RAG-1−/− infected mice.

Expression of IL-10 mRNA was determined by real time PCR in purified splenic CD4^+^ T cells obtained on days 1, 3, 5 and 7 post-infection from wild type (WT) C57/BL6 mice and plasma levels of IL-10 were determined by ELISA on days 1, 3, 5 and 7 pi from WT and RAG^−/−^ mice. We find that CD4^+^ T cells are a significant source of IL-10 by day 5 of both PyL and PyNL infections, since IL-10 mRNA is significantly upregulated in splenic CD4^+^ cells on days 5 and 7 post-infection compared with cells from uninfected mice (Figure 4A). Furthermore, CD4^+^ T cells (and potentially B cells) may be the major source of IL-10 during infection since plasma IL-10 does not increase above baseline levels in RAG^−/−^ mice (Figure 4B, C) except on day 3 pi of PyL infection.

**Figure 5:**
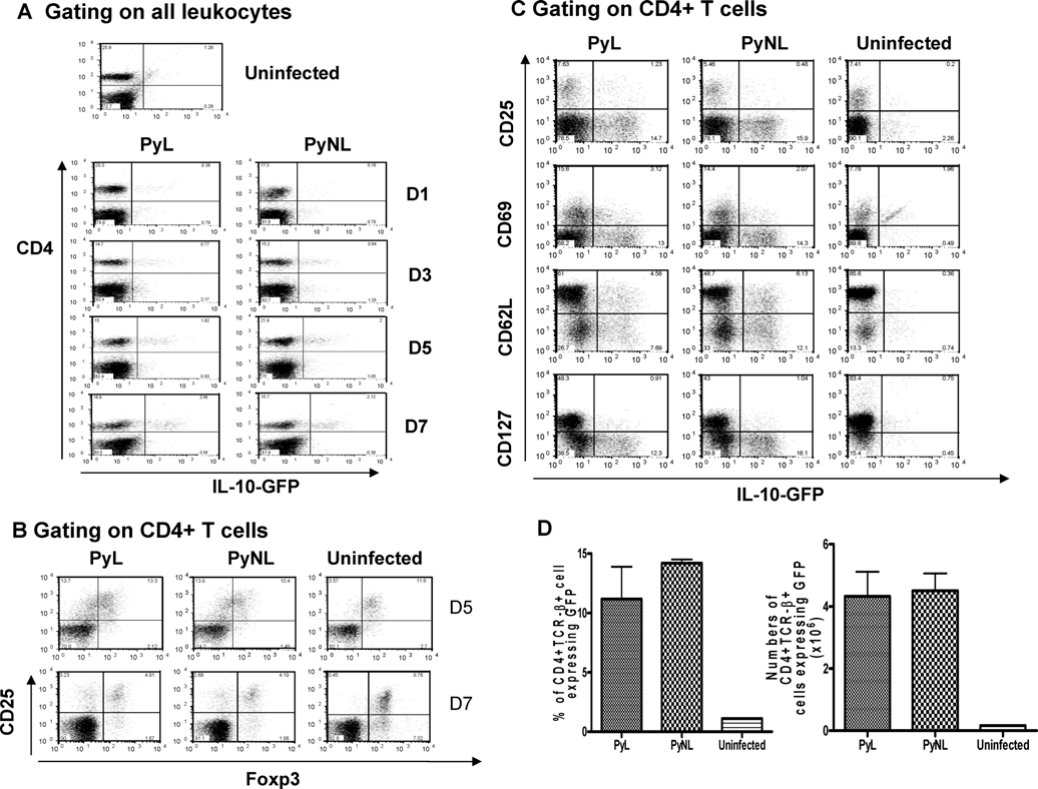
CD127^low^ Foxp3^−^ CD4+ T cells that do not constitutively express CD25 are the major source of IL-10 during *P. yoelii* infection. Transgenic, IL-10-GFP knockin tiger mice were infected with PyL or PyNL. (A) Splenic lymphocytes from infected or uninfected control mice were analysed for expression of CD4+ and GFP. (B,C) 7 days post infection, splenic CD4^+^ T cells were analysed for (B) expression of CD25 and Foxp3, or (C) GFP (IL-10) and CD25, CD69, CD62L or CD127. (D) The frequency and number of GFP+ (IL-10+) CD4^+^ T cells was calculated 7 days post infection in infected or uninfected mice. Groups consisted of 3–5 mice and the results are representative of 2 independent experiments.

To more accurately determine the cellular source of IL-10 during *P. yoelii* infection, splenocytes from IL-10-GFP reporter mice [21] were examined for expression of GFP and various cell surface markers on selected days after PyL or PyNL infection (Figure 5A–C). In both infections, from day 5 onwards, the vast majority of the IL-10^+^ cells were CD4^+^ lymphocytes. At no point during either PyL or PyNL infection did we observe significant IL-10 production by myeloid (CD11b^+^), lymphoid dendritic cells (CD11c^+^) or macrophages (F4-80^+^) (results not shown). IL-10 production by CD19^+^ B cells was observed, on day 7 post-infection, only during PyL but not PyNL infection (results not shown). Moreover, IL-10 producing non-CD4^+^ T cells produced only low quantities of IL-10, whereas CD4^+^ T cells were heterogeneous in their ability to produce IL-10 (Figure 5A).

Since it is not possible to stain for intranuclear Foxp3 without quenching the fluorescence of GFP, IL-10/GFP^+^ CD4^+^ T cells were analysed for expression of CD25, CD62L and CD127 and separately analysed for CD25 and Foxp3 (Figure 5B, C). On day 5 post-infection, IL-10^+^ CD4^+^ T cells showed very variable expression of CD25 with approx. 60% being CD25^−^, indicating that they are not a typical nTreg population. As we have previously observed transient upregulation of CD25 on CD4^+^Foxp3^−^ T cells at this time (Figure 5B and [22]), we considered it likely that at 5 days post-infection the majority of IL-10^+^ cells were Foxp3^−^. In confirmation of this, by day 7 post-infection, IL-10^+^ CD4^+^ T cells were almost exclusively CD25^−^ indicating that, since the majority of Foxp3^+^ cells maintain CD25 expression during *P. yoelii* infection (Figure 5B), CD25^−^Foxp3^−^ CD4^+^ T cells are the primary source of IL-10 during both PyL and PyNL infection. Interestingly the frequencies and numbers of IL-10^+^ CD4^+^TCR-β^+^ cells were equivalent in PyL and PyNL infected mice on day 7 post-infection (Figure 5D).

IL-10^+^ cells were heterogeneous in terms of expression of CD62L suggesting that they comprise of a mixed population of cells in terms of memory/activation status, and despite being Foxp3^−^, the majority of IL-10^+^ CD4^+^ cells were CD127^−^, suggesting that down-regulation of IL-7Rα may be a useful marker for differentiating adaptive Treg from other antigen-experienced T cells (Figure 5C).

### CD4^+^ T cells that produce IL-10 during malaria infection are not classical nTreg, Th1, Th2, or Th17 cells

**Figure 6:**
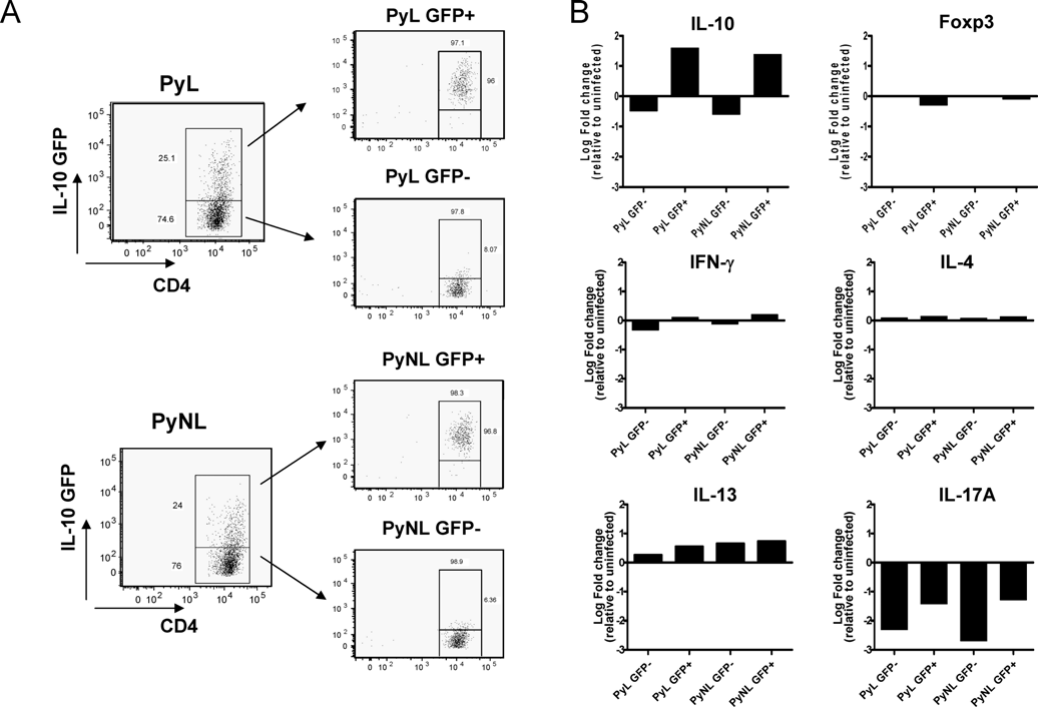
IL-10 producing CD4+ T cells that develop during *P. yoelii* infection are Tr1 cells and not Th1, Th2, or Th17 cells. Splenic CD4^+^ T cells were isolated from transgenic IL-10-GFP mice on day 7 of PyL or PyNL infection and were purified by flow cytometric cell sorting into GFP^+^ (IL-10^+^) and GFP^−^ (IL-10^−^) populations. (A) shows the purity of the purified GFP^+^ and GFP^−^ populations. (B) Expression of IL-10, Foxp3, IFN-γ, IL-4, IL-13 and IL-17A mRNA was determined by real time PCR (Taqman) relative to the house keeping gene, GAPDH. The results are shown as the fold change in expression relative to uninfected naïve CD4+ T cells. For Taqman analysis, purified cells from several mice in each group were pooled.

We have shown that CD4^+^ T cells are the primary source of IL-10 during malaria infection, and that these cells do not express CD25, suggesting that they may not be conventional nTreg cells. Since IL-10 can be produced by various effector CD4^+^ T cell subsets (including Th1, Th2 and Th17 cells), as well as specialised regulatory populations such as Tr1 [19, 20 34–36], we examined the expression of Th1, Th2 and Th17 lineage-associated cytokines in IL-10-producing (GFP^+^) and IL-10-GFP^−^ CD4^+^ T cells purified from IL-10-GFP reporter mice on day 7 of infection. As seen previously (Figure 5), GFP expression was similar in CD4^+^ T cells isolated from PyL and PyNL infected mice (Figure 6A). As expected, IL-10 mRNA was expressed at much higher levels in GFP^+^ than in GFP^−^ cells but cells isolated from PyL and PyNL infected animals expressed similar levels of IL-10 mRNA (Figure 6B). Importantly, Foxp3 mRNA was not upregulated in IL-10-GFP^+^ cells isolated during either PyL or PyNL infection, confirming that the IL-10-producing CD4^+^ T cells that develop during *P. yoelii* infection are neither natural nor induced Foxp3^+^ regulatory T cells. Moreover, GFP^+^ cells did not express significant amounts of mRNA for IFN-γ, IL-4 or IL-17, thus distinguishing them from classical Th1, Th2 and Th17 cells. Although IL-10-GFP^+^ cells expressed IL-13 mRNA, levels were comparable to those seen in GFP^−^ cells indicating that IL-10 producing cells did not preferentially co-produce IL-13. Thus, the IL-10-producing CD4^+^ T cells induced during *P. yoelii* infection fit the definition [35] of adaptive, Tr1, regulatory T cells.

### IL-10 from CD4^+^ T cells inhibits parasite killing during infection

**Figure 7:**
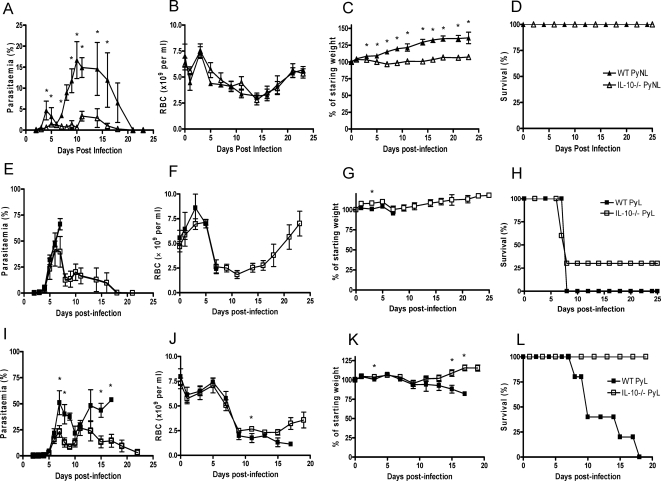
IL-10 impedes parasite clearance during both PyL and PyNL infection. The course of infection with (A–D) PyNL and (E–L) PyL in mice given (A–H) 10^4^ pRBC's or (I–L) 10^3^ pRBC's was compared in WT and IL-10^−/−^ mice by monitoring (A, E, I) parasitaemia (B, F, J) anaemia, (C, G, K) weight loss and ( D, H, L)survival. Groups consisted of 4–5 mice and the results are representative of 4 independent experiments. * indicates significant differences (p<0.05) between WT and IL-10−/− infected mice.

To determine whether IL-10 production from T cells is functionally important during Py infection, we first compared the course of PyL and PyNL infection in IL-10^−/−^ and WT mice (Figure 7). PyNL infection was significantly attenuated - with significant reductions in parasitaemia and anaemia in IL-10^−/−^ mice compared with WT mice (Figure 7A–D), although the IL-10^−/−^ mice did lose significantly more weight than age-matched WT mice (Figure 7C). Furthermore, approx 30% (6/21 mice) of IL-10^−/−^ (but not WT) mice infected with 10^4^ PyL pRBC were able to control their infections and survived (Figure 7E–H), with parasitaemia declining from a peak of approx 45% on day 6pi. Moreover, IL-10^−/−^ (but, again, not WT) mice given a low dose PyL infection (10^3^ pRBC) were fully able to control parasitaemia and 100% of the mice survived (Figure 7I–L).

Taken together, these data indicate that IL-10 suppresses immune effector mechanisms which would otherwise be able to control low dose PyL infections. Since this IL-10 emanates principally from CD4^+^ T cells (Figure 5) we hypothesised that IL-10-deficient CD4^+^ T cells may promote more effective parasite control than WT CD4^+^ T cells. To test this, purified naïve WT or IL-10^−/−^ CD4^+^ T cells were adoptively transferred into RAG^−/−^ mice which were then infected with PyNL or PyL parasites.

**Figure 8:**
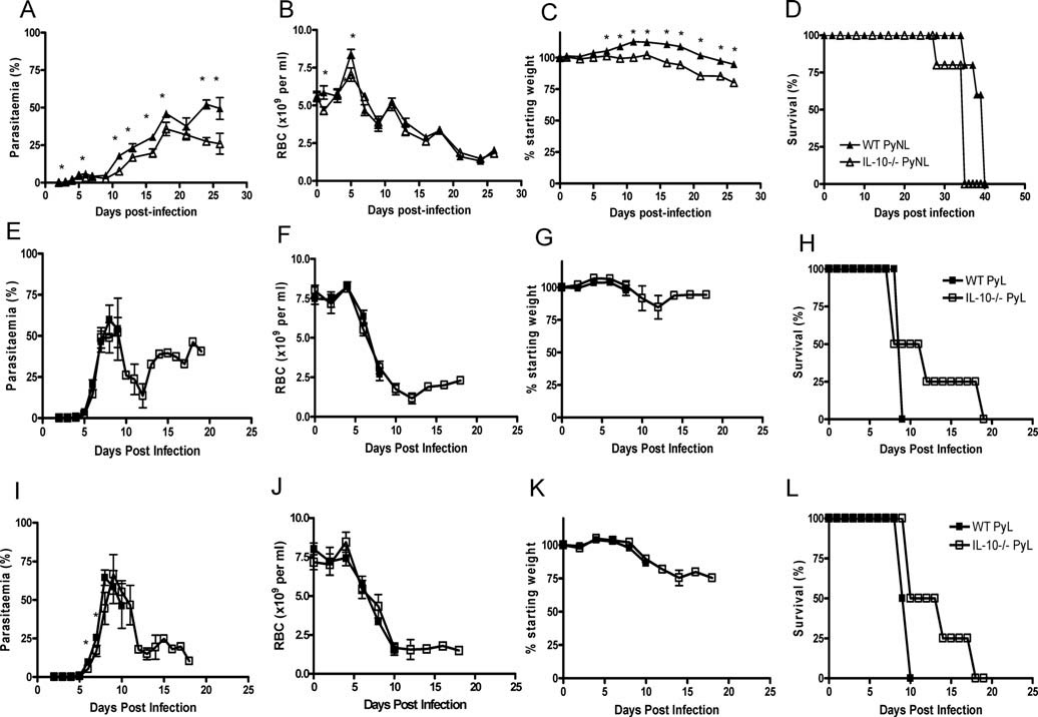
T cell-derived IL-10 inhibits parasite clearance during PyL and PyNL infections. Prior to infection with (A–D) PyNL and (E–L) PyL with (A–H) 10^4^ or (I–L) 10^3^ pRBC's, RAG-1^−/−^ mice received naïve CD4^+^ T cells that had been purified from either WT or IL-10^−/−^ mice by magnetic bead sorting. The course of infection was followed by monitoring (A, E, I) parasitaemia (B, F, J) anaemia and (C, G, K) weight loss and (D, H, L) survival. Groups consisted of 4 mice and the results are representative of 2 independent experiments. * indicates significant differences (p<0.05) between mice receiving WT and IL-10−/− CD4 T cells.

PyNL-infected RAG^−/−^ mice that had received IL-10^−/−^ CD4^+^ T cells developed significantly lower parasite burdens than those which had received WT CD4^+^ T cells (Figure 8A). Although both groups developed similar levels of anaemia, mice that received IL-10^−/−^ T cells lost significantly more weight and succumbed to infection more rapidly than mice that received WT CD4+ T cells (Figure 8B–D). Exacerbation of disease despite improved parasite control in mice receiving IL-10^−/−^ CD4^+^ T cells was associated with more extensive proliferation of the adoptively transferred T cells (IL-10^−/−^ T cells comprised >30% of total splenic leucocytes compared with <10% for transferred WT cells), higher concentrations of circulating IFN-γ and lower plasma concentrations of IL-10 (data not shown). These data are consistent with the conclusion that recipients of IL-10^−/−^ CD4^+^ T cells died of immunopathology whilst recipients of WT CD4^+^ T cells eventually died because they were unable to fully resolve their infections.

By contrast, RAG^−/−^ mice that had received IL-10^−/−^ CD4^+^ T cells were somewhat better able to control infections with 10^3^ (8E–H) or 10^4^ (8I–L) PyL infections than were mice receiving WT CD4^+^ T cells; a proportion of mice receiving IL-10^−/−^ T cells were able to control their infections, although failure to fully eliminate parasites eventually led to death from anaemia. Thus, IL-10 derived from CD4^+^ T cells significantly modulates the outcome of both PyL and PyNL infection.

### IL-10 protects against immune pathology during *P. yoelii* infection

It has previously been shown that IL-10^−/−^ mice succumb to normally avirulent *P. chabaudi chabaudi* infections despite comparable - or more effective - control of malaria parasitaemia compared to WT mice [9]. The increased susceptibility of IL-10^−/−^ mice is due to elevated plasma concentrations of IFN-γ and TNF-α [10] and survival of IL-10^−/−^ mice following malaria infection can be enhanced by treatment with anti-TNF-α [10]. Whilst there was no marked difference in mortality between *P. yoelii*-infected IL-10^−/−^ and WT mice, IL-10^−/−^ mice (and RAG^−/−^ mice reconstituted with IL-10^−/−^ T cells) lost significantly more weight than mice reconstituted with WT T cells during PyNL infection, indicative of more severe morbidity (Figure 7C, 8C).

**Figure 9:**
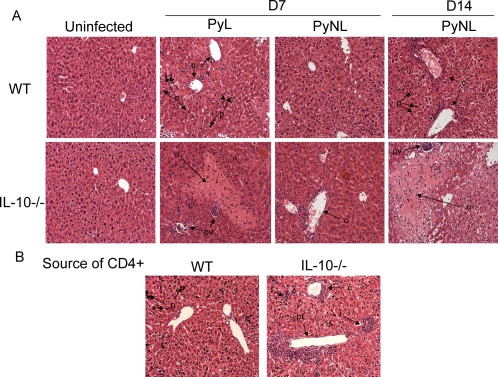
IL-10 ameliorates hepatic pathology during PyL and PyNL infection. Liver pathology was examined (A) in WT and IL-10^−/−^ mice that were either uninfected or had been infected with *P. yoelii* 7 days (PyL, PyNL) or 14 days (PyNL) previously, (B) on day 25 post-infection with PyNL in RAG-1^−/−^ mice reconstituted with either WT or IL-10^−/−^ CD4+ T cells prior to infection. Groups consisted of 4–5 mice and the slides shown are representative of mice from 2 independent experiments. Arrows highlight areas of interest: c = central vein periportal infiltration, pv = vessels packed with inflammatory cells, p = pigmented kupffer cells, n = necrosis, i = inflammation in parenchymia, pt = necrosis and gross infiltration in portal triad.

Histopathological examination of infected animals did not reveal any liver or lung damage 3 days post-infection (data not shown) but revealed significantly more hepatic cellular changes including periportal inflammation, necrosis and bridging necrosis in IL-10^−/−^ mice than in WT mice on days 7 and 14 post-infection (Figure 9A) and this was significantly more severe in PyL-infected than PyNL-infected animals on day 7 post-infection. We also found that by day 25 of PyNL infection, RAG^−/−^ recipients of IL-10^−/−^ CD4^+^ T cells had developed significantly more severe hepatic periportal inflammation and necrosis (including bridging necrosis) than RAG^−/−^ recipients of WT CD4^+^ T cells (Figure 9B). Thus, T cell derived IL-10, although negatively regulating parasite killing, is protective during malaria infection by preventing the onset of immunopathology.

## Discussion

It is well established, in a variety of infections, that regulatory cytokines both ameliorate immunopathology and delay pathogen clearance [5], [8], [9]–[11], [37]–[42]. Manipulation of these cytokines by vaccination or immunotherapy, to simultaneously enhance pathogen clearance and limit the associated pathology, requires a better understanding of their cellular sources and mechanisms of induction. Important roles have been demonstrated for both IL-10 [6]–[11] and TGF-β [4],[5] in modulating the outcome of murine malaria infections, and observational data strongly suggests that they play a similar role in human infections [43]–[45]. Recently, endogenous or natural, CD25^hi^, Foxp3^+^ CD4^+^ T cells (nTreg) have been implicated as major regulators of malarial pathology [13],[46] but their mechanisms of action remain undefined. Attempting to elucidate the role of nTreg in murine *Plasmodium yoelii* infections, we were surprised to find no role for these cells in regulating the outcome of either high dose (10^4^) or lower dose (10^3^) lethal (Py17XL; PyL) or non-lethal (Py17X; PyNL) infection in either C57BL/6 or BALB/c mice. In contrast, we find that adaptive, IL-10-producing CD4^+^ Tr1 cells (CD25^−^, Foxp3^−^, CD127^−^, IFN-γ^−^, IL-4^−^ and IL-17^−^), are generated during both PyL and PyNL infections and are associated with down-regulation of pro-inflammatory responses, moderation of both morbidity and mortality and failure to clear parasites. Crucially, we were able to demonstrate a causal relationship between these various observations by showing that IL-10^−/−^ CD4^+^ T cells adoptively transferred into RAG^−/−^ mice provided more effective parasite control than did WT CD4^+^ T cells, but at the cost of more severe pathology.

We conclude that induced Foxp3^−^ regulatory T cells, characterised by down-regulation of CD127/IL-7Rα, modulate the inflammatory response to *Plasmodium yoelii* malaria by production of IL-10. Although it has been observed in humans [47],[48] and mice [48] that CD127 is down-regulated on endogenous (Foxp3^+^) regulatory T cells, our data demonstrate – for the first time - that CD127 is also down-regulated on adaptive (Foxp3^−^) Tr1 cells.

The lack of any effect of anti-CD25 antibody treatment on the course of PyL infection in our experiments contradicts the published data [13],[46]. Despite numerous attempts, using three different depletion protocols - including a protocol identical to that previously reported to ameliorate PyL infection [13], in both C57BL/6 and BALB/c mice we were unable to reproduce the published observations. Anti-CD25 treatment failed to ameliorate PyL infection initiated by a 10 fold lower dose of parasites, discounting the possibility that the virulence of high dose PyL infection masks regulatory activity of nTreg that would otherwise be evident during a less virulent infection. Furthermore, adoptive transfer of CD25^+^ and/or CD25^−^ CD4^+^ T cells into RAG^−/−^ mice also failed to reveal any role for CD25^+^ Foxp3^+^ cells in this infection. The discrepancy between our findings and those of other labs is reminiscent of the disparate results obtained for *P. berghei* ANKA infection which report that depletion of CD25^+^ regulatory cells either facilitates parasite control and prevents the onset of the cerebral pathology infection in C57BL/6 mice [49], or enhances effector T cell responses and increases the severity of brain pathology in normally resistant BALB/c mice [50] or has no effect on cerebral pathology [51]. One explanation for these inconsistent results may be differences in prior exposure to pathogens or commensal organisms between mice in different laboratories. Components of the normal intestinal flora of conventionally housed animals are essential for development of intestinal nTreg [52] and nTreg development is facilitated by the presence of covert infections such as *Helicobacter hepaticus* [53]. Depletion of nTreg by anti-CD25 treatment in such mice may lead to more profound alteration in the effector / regulatory cell balance than in mice (such as those used in our studies) raised in low-infection environments.

Although, we could show no role for nTreg in acute PyL and PyNL infection, we have shown that the adaptive IL-10-producing regulatory T cells that develop during *P. yoelii* infection hinder parasite control but simultaneously limit disease severity. In contrast to recent studies describing a role for IL-10 producing Th1 cells in *Toxoplasma gondii* [20] and *Leishmania spp* [19],[54],[55] infections, the adaptive IL-10 producing Tregs we describe do not co-express IFN-γ or other effector cytokines and better fit the definition of Tr1 cells. Nevertheless in several virulent protozoal infections (PyL, *Toxoplasma gondii*, *Leishmania major* SD and *L. donovani*), adaptive IL-10-producing CD4^+^ T cells are required to regulate the fulminant Th1-effector responses that are induced whereas classical (Foxp3^+^) Treg appear to be sufficient to regulate the effector response to a less virulent (healing) strain of *L. major* [15]. Given that naïve CD4^+^ T cells can develop into adaptive Treg after interaction with IL-10-producing dendritic cells expressing low levels of co-stimulatory molecules [56]–[59], it is possible that the induction of Treg during *P. yoelii* infection is linked to the modulation of macrophage and dendritic cell function that occurs in response to prolonged toll-like receptor signalling [60]. Alternatively, parasite-induced TGF-β [40],[60], IL-6 [44] and/or IL-27 may synergise to promote production of IL-10 by Th1, Th2, Th17 and Tr1 cells [34],[36],[61],[62].

We have not yet definitively identified the mechanism by which IL-10 suppresses parasite clearance but given our recent findings that control of the acute phase of *P. yoelii* parasitaemia is critically dependent on macrophages [22], it is likely that T cell-derived IL-10 acts directly on macrophages to inhibit their anti-parasitic mechanisms. It is also possible that, as in mycobacterial infections, adaptive Treg induce an autocrine signalling loop in which macrophages both secrete and respond to IL-10 with consequent down regulation of effector function and pathology via a STAT-3 –dependent pathway [63]–[66].

In summary, we have demonstrated that adaptive, but not natural, regulatory T cells control parasite numbers during PyL and PyNL infections whilst also limiting the onset of immunopathology. These cells are characterised by lack of expression of CD25 and Foxp3, down-regulation of CD127 and production of IL-10 but not IFN-γ, IL-4 or IL-17. Taken together with our data highlighting the importance of macrophages in the control of malaria infection [22], these findings identify an important pathway of adaptive, T cell- mediated control of innate immune responses. Further studies are required to identify the pathways leading to induction of this important regulatory cell population.

## Materials and Methods

### Mice and parasites

C57BL/6, Foxp3-GFP (F2: 129/C57BL/6; from A. Rudensky, University of Washington, 24), C57BL/6 RAG-1^−/−^, C57BL/6 IL-10^−/−^ and BALB/c mice were bred in-house or purchased from Harlan and maintained under barrier conditions at LSHTM. IL-10-GFP reporter mice [21] were maintained under barrier conditions at the National Institutes of Health. Cryopreserved *Plasmodium yoelii* 17X (non lethal; PyNL) and *P. yoelii* 17XL (lethal; PyL) parasites were passaged once through mice before being used in experimental animals.

Unless stated otherwise, male or female mice, 7–9 weeks of age, were infected intraveneously with 1 × 10^3^ or 1 × 10^4^ parasitised red blood cells (pRBC). Parasitaemia was determined daily by examination of Giemsa-stained thin smears of tail blood for the first seven days of infection and every second day thereafter. On every second day, mice were weighed and RBCs were counted using an automated haemoanalyser (Beckman Coulter). Plasma was stored (at −20°C) for cytokine quantification. On selected days post-infection, mice were sacrificed and spleens were removed. Single spleen cell suspensions were prepared by homogenisation through a 70 µm cell strainer (BD Biosciences) and live cells enumerated by trypan blue exclusion.

### Cell sorting

CD4^+^ T cells were positively selected using anti-mouse CD4-conjugated midiMACS beads (Miltenyi Biotec) according to the manufacturer's instructions and the purity of eluted cells was checked by flow cytometry. In some experiments, the CD4+ cells were labelled with anti-mouse CD4 (GK1.5: Rat IgG2b: E-bioscience) and anti-mouse CD25 (PC61: Rat IgG1: E-bioscience) fluorochrome-labelled antibodies and sorted, using a BD FACSVantage SE, into CD4^+^CD25^+^ and CD4^+^CD25^−^ populations. In separate experiments CD4^+^ T cells, isolated from IL-10-GFP reporter mice [21] on day 7 of infection, were labelled with anti-mouse CD4 (GK1.5: Rat IgG2b: E-bioscience) and IL-10 producing (GFP^+^) and non-IL-10 producing (GFP^−^) CD4^+^ T cells were purified by flow cytometric cell sorting.

### Real time PCR

IL-10, Foxp3, IFN-γ, IL-4, IL-13 and IL-17A mRNA were quantified by Taqman (ABI, Warrington, UK). RNA was extracted (RNAeasy) and DNAse1 treated prior to cDNA synthesis. cDNA expression for each sample was standardised using the housekeeping gene GAPDH. Cycling conditions were: initialisation 2 min at 50°C and 10 min at 95°C followed by 40 cycles of 15 sec at 95°C and 1 min at 60°C.

Primer sequences:

IL-10 Forward ATGCTGCCTGCTCTTACTGACTG

 Reverse CCCAAGTAACCCTTAAAGTCCTGC,

Foxp3 Forward CACCTATGCCACCCTTATCC,

 Reverse CGAACATGCGAGTAAACCAA

IFN-γ Forward AGA GCC AGA TTA TCT CTT TCT ACC TCA G

 Reverse CCT TTT TCG CCT TGC TGT TG

IL-4 Forward ACG AGG TCA CAG GAG AAG GGA

 Reverse AGC CCT ACA GAC GAG CTC ACT C

IL-13 Forward CCTCTGACCCTTAAGGAGCTTAT

 Reverse CGTTGCACAGGGGAGTCT

IL-17A Forward TGTGAAGGTCAACCTCAAAGTC

 Reverse AGGGATATCTATCAGGGTCTTCATT

### Cytokine ELISA

Rat anti-mouse IL-10 (JES5-2A5; Rat IgG_1_; Mabtech, Sweden) or rat anti-mouse interferon (IFN)-γ (AN-18; Rat IgG1; eBioscience) antibodies were used as capture antibodies, diluted in 0.5 M Tris-HCl, pH 8.9 buffer. Biotinylated rat anti-mouse IL-10 MAb (JES5-16E3; Rat IgG_2b_; Mabtech) or rat anti-mouse IFN-γ MAb (R4-6A2; Rat IgG1; Mabtech) were used as detecting antibodies and were visualised using streptavidin-alkaline phosphatase (eBioscience) and *p*-nitrophenyl phosphate (Sigma Aldrich, UK). Absorbance was read at 405 nm using a MRX TC II microplate reader (Dynex Technologies Ltd,. UK)

### Flow cytometry

For flow cytometric analysis, cells were surface stained with anti-mouse CD4 (RM4-5; Rat IgG_2a_; BD Biosciences), anti-mouse CD25 (PC61; Rat IgG_1_; eBioscience), anti-mouse CD69 (H1.2F3; Armenian Hamster IgG; eBioscience), anti-mouse CD62L (MEL-14; Rat IgG2a; eBioscience) or anti-mouse CD127 (A7R34; Rat IgG2a; eBioscience). Intracellular Foxp3 staining using anti-mouse Foxp3 (FJK-16s; Rat IgG_2a_; eBioscience) was performed by permeabilising cells with 0.1% Saponin/PBS. Cells were concurrently incubated with anti-mouse CD16/32 (Fc block) when staining with all conjugated antibodies. Flow cytometric acquisition was performed using a FACSCalibur (BD Immunocytometry Systems, USA) and all analysis was performed using Flowjo software (Treestar Inc., OR, USA)

### Histopathology

Liver and lung tissues were fixed in 10% Formalin-saline. Fixed tissues were paraffin embedded and stained with haematoxylin and eosin. Slides were microscopically examined at 20X magnification.

### Statistical analysis

Statistical significance was determined using Student's T test, unless otherwise stated, with P<0.05 taken as indicating a significant difference.

## Supporting Information

Figure S1 Anti-CD25 antibody administration does not affect the outcome of low dose PyL infection. C57BL/6 mice were treated with either a single dose of 0.75 mg 7D4, or with 0.25 mg 7D4 combined with 0.75 mg PC61, 3 days prior to infection with 10^3^ PyL parasites. The course of PyL in anti-CD25 antibody-treated mice and control mice was then followed by monitoring (A) parasitaemia, (B) anaemia and (C) mortality. # indicates significant differences (P<0.05) between 7D4 and 7D4 + PC61 treated mice. No significant differences were observed between the other groups of mice. Groups consisted of 4 mice and the results are representative of 2 independent experiments.
(0.02 MB TIF)

Figure S2 Anti-CD25 antibody administration does not affect the outcome of PyNL infection. C57BL/6 mice were treated with either a single dose of 1 mg PC61 7 days prior to infection with PyNL (Expt 1) or with a single dose of 0.75 mg 7D4 or 0.25 mg 7D4 combined with 0.75 mg PC61 on the day of PyNL infection (Expt 2). The course of PyNL in anti-CD25 antibody-treated mice and control mice was then followed by monitoring (A, D) parasitaemia, (B, E) anaemia and (C, F) weight loss. No mortality occurred in any of the groups during either experiment. * indicates significant differences (P<0.05) between PC61 treated and control mice. No significant differences were observed between 7D4, 7D4 + PC61 treated mice and control mice. Groups consisted of 3–5 mice per group.
(0.02 MB TIF)

Figure S3 Anti-CD25 antibody administration does not affect the outcome of PyL infection in BALB/c mice. BALB/c mice were treated with either a single dose of 0.75 mg 7D4, or with 0.25 mg 7D4 combined with 0.75 mg PC61, 3 days prior to infection with 104 PyL pRBC or with 3 repeated injections of 0.5 mg 7D4 on days −3, −1 and +5 relative to PyL infection. The efficiency of depletion of CD25+ and Foxp3+ CD4+ T cells was determined in peripheral venous blood by measuring the expression of CD25^+^ and Foxp3^+^ CD4^+^ T cells was determined in peripheral venous blood by measuring the expression of (A) CD25 and (B) Foxp3 on CD4^+^ T cells. The course of PyL in anti-CD25 antibody-treated mice and control mice was then followed by monitoring (C) parasitaemia, (D) anaemia and (E) mortality. Symbols represent significant differences (p<0.05) between groups: ∼ 7D4 vs PBS, Φ 7D4/PC61 vs PBS, + 7D4/PC61 vs 7D4 x3, # 7D4 vs 7D4/PC61. Groups consisted of 5 mice.
(0.15 MB TIF)
