# Supplementary figures and images for: IL-10 from CD4+CD25−Foxp3−CD127− Adaptive Regulatory T Cells Modulates Parasite Clearance and Pathology during Malaria Infection
Source: PLoS Pathog. 2008 Feb 29;4(2):e1000004. doi: 10.1371/journal.ppat.1000004 (PMC2291447; doi:10.1371/journal.ppat.1000004)

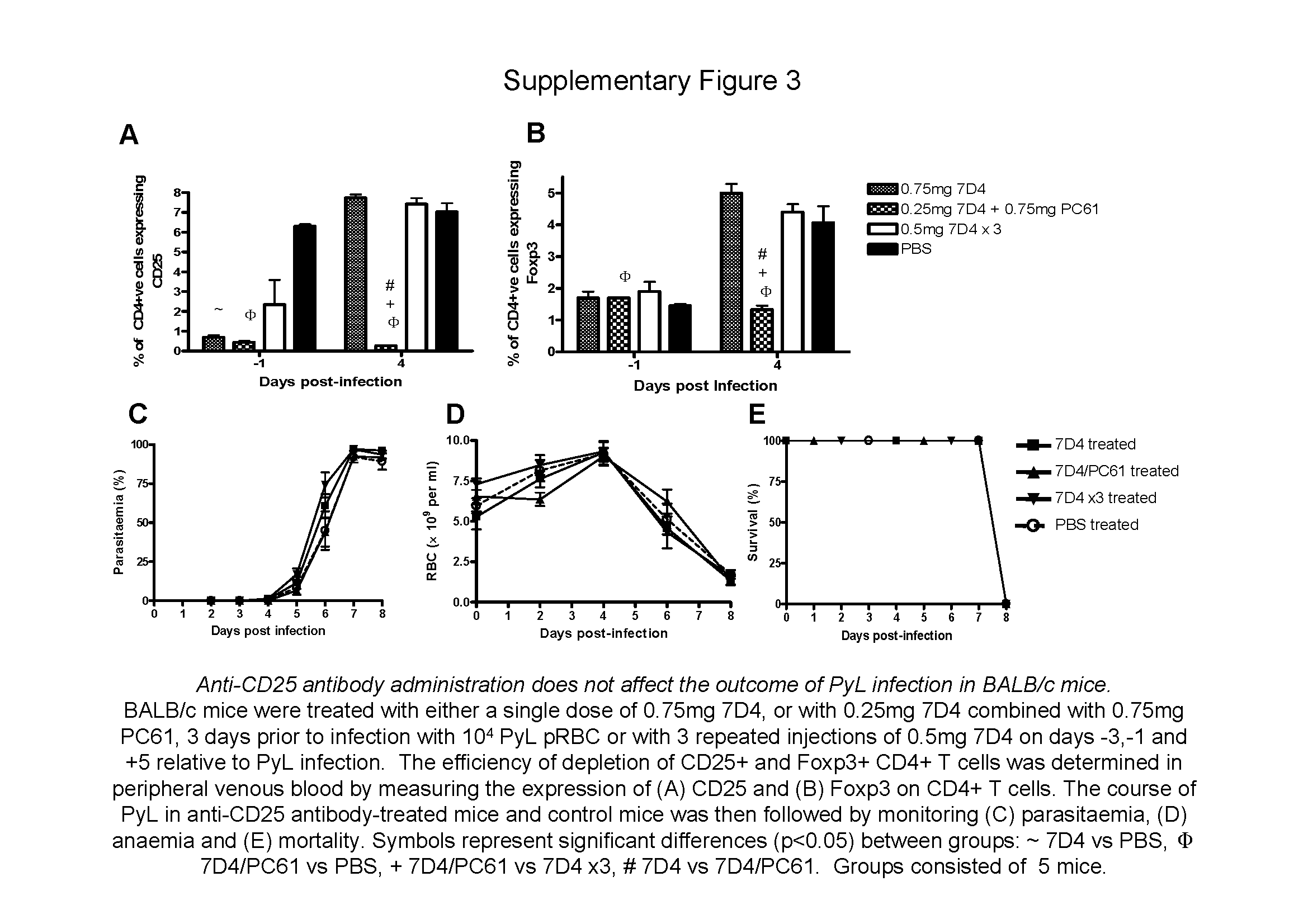

Supplement: Figure S3 — BALB/c mice were treated with either a single dose of 0.75 mg 7D4, or with 0.25 mg 7D4 combined with 0.75 mg PC61, 3 days prior to infection with 104 PyL pRBC or with 3 repeated injections of 0.5 mg 7D4 on days −3, −1 and +5 relative to PyL infection. The efficiency of depletion of CD25+ and Foxp3+ CD4+ T cells was determined in peripheral venous blood by measuring the expression of CD25+ and Foxp3+ CD4+ T cells was determined in peripheral venous blood by measuring the expression of (A) CD25 and (B) Foxp3 on CD4+ T cells. The course of PyL in anti-CD25 antibody-treated mice and control mice was then followed by monitoring (C) parasitaemia, (D) anaemia and (E) mortality. Symbols represent significant differences (p<0.05) between groups: ∼ 7D4 vs PBS, Φ 7D4/PC61 vs PBS, + 7D4/PC61 vs 7D4 x3, # 7D4 vs 7D4/PC61. Groups consisted of 5 mice. (0.15 MB TIF) [file ppat.1000004.s003.tif]
